# Construction and Analysis of a New Resting-State Whole-Brain Network Model

**DOI:** 10.3390/brainsci14030240

**Published:** 2024-02-29

**Authors:** Dong Cui, Han Li, Hongyuan Shao, Guanghua Gu, Xiaonan Guo, Xiaoli Li

**Affiliations:** 1 Hebei Key Laboratory of Information Transmission and Signal Processing, Yanshan University, Qinhuangdao 066004, China; lihanxtky@163.com (H.L.); 15567505275@163.com (H.S.); guguanghua@ysu.edu.cn (G.G.); guoxiaonan@ysu.edu.cn (X.G.); 2 School of Information Science and Engineering, Yanshan University, Qinhuangdao 066004, China; 3 National Key Laboratory of Cognitive Neuroscience and Learning, Beijing Normal University, Beijing 100875, China; xiaoli@bnu.edu.cn

**Keywords:** whole-brain network model, neural mass model, Wendling model, EEG, brain network simulation

## Abstract

Background: Mathematical modeling and computer simulation are important methods for understanding complex neural systems. The whole-brain network model can help people understand the neurophysiological mechanisms of brain cognition and functional diseases of the brain. Methods: In this study, we constructed a resting-state whole-brain network model (WBNM) by using the Wendling neural mass model as the node and a real structural connectivity matrix as the edge of the network. By analyzing the correlation between the simulated functional connectivity matrix in the resting state and the empirical functional connectivity matrix, an optimal global coupling coefficient was obtained. Then, the waveforms and spectra of simulated EEG signals and four commonly used measures from graph theory and small-world network properties of simulated brain networks under different thresholds were analyzed. Results: The results showed that the correlation coefficient of the functional connectivity matrix of the simulated WBNM and empirical brain networks could reach a maximum value of 0.676 when the global coupling coefficient was set to 20.3. The simulated EEG signals showed rich waveform and frequency-band characteristics. The commonly used graph-theoretical measures and small-world properties of the constructed WBNM were similar to those of empirical brain networks. When the threshold was set to 0.22, the maximum correlation between the simulated WBNM and empirical brain networks was 0.709. Conclusions: The constructed resting-state WBNM is similar to a real brain network to a certain extent and can be used to study the neurophysiological mechanisms of complex brain networks.

## 1. Introduction

Mathematical modeling and computer simulation are the main methods for understanding neural mechanisms, especially in the field of computational neural systems. A whole-brain network model (WBNM) is a network model that is composed of coupled brain regions; these are usually represented by a neural mass model (NMM), which represents the average activity of a brain region on a mesoscopic scale. Their interconnection is usually based on biologically informed data on the brain’s structural connectivity [1]. In this study, our aim was to use mathematical modeling methods to construct a WBNM with neurophysiological significance. It is similar to real human brains in terms of brain rhythm, structural connectivity, functional connectivity, and brain network characteristics, and the complexity of the model is also considered. This enables researchers to study the neurophysiological mechanisms of functional diseases of the brain and brain modulations based on this model.

The human brain is a multiscale complex system composed of many dynamic units that interact to generate various behavioral functions [2]. From a small-scale perspective, the entire brain is composed of numerous types of neurons that are connected to one another, and electrical pulses propagate through this vast distributed network. From a large-scale perspective, the entire brain can be seen as a system network with complex dynamic characteristics. At a mesoscopic scale, that is, at the level of the neural masses or brain regions, the collective activity often exhibits rhythmic patterns [3]. Neural oscillations promote time coordination in distant regions, leading to the phenomenon of functional interactions, which play a crucial role in interregional communication and information transmission between distributed brain regions [4,5,6]. Therefore, a WBNM can help researchers study the functional connectivity of EEG signals and the potential mechanisms of the brain from both mesoscopic and macroscopic perspectives. 

In order to construct a WBNM, we need to select a model as a node. An NMM consists of a series of differential equations and provides a new way to study the brain’s rhythmic activity and the neural mechanisms of functional diseases of the brain [7]. A key strength of NMMs over alternative statistical mathematical frameworks lies in their foundation on anatomical and neurophysiological principles with state variables and parameters that possess well-defined physiological interpretations. This feature provides a substantial correspondence between the mathematical model and the actual EEG signal, and it makes the use of NMMs more reliable when analyzing actual EEG signals [8,9,10,11,12]. Freeman [13] initiated the “neural mass” concept by aggregating the activities of neuronal groups into a lumped parameter framework, while its mathematical underpinnings were elaborated by Wilson and Cowan [14]. Subsequently, Lopes da Silva et al. [15] introduced the alpha rhythm model (ARM), which integrated two thalamic neuronal populations to mimic thalamic alpha rhythms, underscoring the thalamus’s critical function in conveying sensory information to the cerebral cortex [16]. On this foundation, Jansen and Rit [17] developed the more sophisticated cortical NMM, which was termed the J&R model and was aimed at modeling the visual cortex’s responses to visual stimuli. This model is distinguished by its ability to produce alpha waves using three distinct neuronal populations: pyramidal cells and both excitatory and inhibitory interneurons. Further extending this framework, Wendling et al. [18] incorporated an additional fast inhibitory interneuron class into the J&R model, categorizing the inhibitory neurons into slow and fast subgroups for enhanced model fidelity. In this study, they chose the Wendling model for the nodes of their WBNM. The following were the main reasons: Compared to simple models that only include excitation inhibition circuits, Wendling models have rich dynamic characteristics and can generate six common EEG signals, including background noise, typical alpha waves, and epileptic spike waves; the Wendling model has been widely used in the study of the cerebral cortex, especially for seizures and the transmission mechanism of epilepsy [19,20,21]; the structure of the Wendling model is relatively simple, and the computational cost of constructing a WBNM is tolerable. Ursino [22] developed a new model by modifying the structure of the fast inhibition circuits of the Wendling model, and it was able to generate gamma waves but also had a correspondingly increased. Therefore, its application is not as widespread as that of the Wendling model.

Anatomical studies have divided the brain into different regions. To simulate the coupling between brain regions, structural connectivity data are typically used to construct WBNMs. In recent years, researchers have conducted some valuable studies on WBNMs and their modeling methods [1,23] and used WBNMs to understand the working mechanisms of the brain [2,24,25,26,27], as well as the neural mechanisms and treatments of functional diseases of the brain [28,29,30,31]. Neurolib is a comprehensive Python framework designed for whole-brain modeling, and it allows researchers to simulate the mesoscopic activity of brain regions through neural mass models [1]. By incorporating structural and functional brain data, including fMRI-derived BOLD signals, it enables precise calibration of models against empirical observations. The framework facilitates the in-depth analysis and optimization of brain models by providing advanced tools for exploring parameter spaces and fitting models to multimodal empirical data. The concept of a Virtual Brain Platform was introduced in [23]. This work established a neuroinformatics platform based on real brain connectivity that can simulate a whole-brain network. Several widely used neurodynamic models are embedded in this platform, and they serve as the basis for inferring neurophysiological mechanisms at different brain scales and ultimately generating macroscopic neuroimaging signals.

Papadopoulos et al. [2] built a large-scale brain model wherein 82 brain areas were represented as Wilson–Cowan neural masses. They investigated how the stimulation of a particular brain area affected the network dynamics. The research results indicated that the network exhibited different responses to regional perturbations based on the baseline state of the system. Through advanced fMRI and diffusion spectrum imaging tractography coupled with computational modeling, Honey et al. [24] uncovered that resting-state functional connectivity in the human cerebral cortex is intricately modulated by its anatomical structure, despite the presence of connections between structurally unlinked regions. These results challenged the feasibility of inferring structural connectivity from functional connectivity, highlighting the role of indirect connections and spatial considerations in shaping functional network dynamics. This study bridges the gap between structural and functional brain networks, offering insights into their complex interplay.

Schirner et al. [25] developed a groundbreaking model that merged EEG-driven neural dynamics with connectome data; they replicated resting-state fMRI signatures and decoded complex neurophysiological relationships. This method bridged multiple observational scales—from oscillatory activity to network topology—advancing our understanding of brain function. Their findings provide a comprehensive framework for interpreting non-invasive brain measurements. Giannakakis et al. [26] leveraged the Wilson–Cowan framework and synaptic plasticity to simulate brain dynamics in real time, enabling studies of long-term changes due to injury or development. This represents a crucial advancement in understanding brain plasticity and recovery. Endo et al. [27] used the Larter–Breakspear model, and they correlated fMRI and EEG/MEG data to uncover the structural influence on resting-state brain dynamics. Their findings pinpoint synchronization patterns that bridge fast EEG microstates with slow fMRI fluctuations, enhancing our grasp of neural connectivity’s role in brain function.

Hann et al. [28] constructed a WBNM with interconnected neural masses, where each neural node was based on the Arm model. The authors used this WBNM to serve as a virtual test ground for developing effective interventions for Alzheimer’s disease. They explored six intervention strategies that adjusted the excitatory or inhibitory levels of neurons, aiming to identify potential methods for delaying the degradation of brain network connectivity. Their findings underscore that selectively boosting excitatory neuron activity emerges as the most efficacious intervention, markedly enhancing network integrity and, thereby, illuminating a promising pathway for therapeutic development aimed at sustaining or rejuvenating the neuronal network in AD. Taylor et al. [29] constructed an epilepsy WBNM based on Benjamin’s epilepsy model. They integrated dynamic computational models with static structural connectivity data from diffusion-weighted MRI, offering a novel approach to mapping epilepsy’s complex neurodynamics. Hutchings et al. [30] utilized a whole-brain epilepsy model tailored to individual structural connectivity profiles from DTI scans of 22 left TLE patients and 39 controls to explore seizure dynamics and surgical outcomes. Kunze et al. [31] applied transcranial direct-current stimulation (tDCS) across a network model of 74 cerebral areas, exploring its effects on brain dynamics and structural connectivity while being guided by the human connectome.

In this study, our objective was to develop a WBNM that more accurately reflects the functional connectivity and structural network organization of the human brain. The Wendling model was used as the node for the WBNM because of its rich spectrum and simple structure. In order to construct the edge of the WBNM, that is, the coupling strength matrix, we used actual MRI data to form an empirical structural connectivity matrix and empirical functional connectivity matrix. But only the relative coupling strength matrix could be obtained by calculating the fiber tracts between region pairs (please refer to Section 2 for specific calculation methods), so an overall multiple was needed to adjust the coupling strength, that is, a global coupling coefficient *C* was used to rescale the structural connectivity strength between regions. We hope that by adjusting the global coupling coefficient *C*, the WBNM can simulate real brain functional connections to a certain extent. Finally, we analyzed the differences between simulated brain networks and actual brain networks at different threshold levels, along with their small-world network properties, using four graph-theoretical measures: the average degree, characteristic path length, clustering coefficient, and global efficiency. We found that the simulated brain networks and actual brain networks exhibited strong similarities in their network characteristics.

## 2. Materials and Methods

Constructing a whole-brain network model (WBNM) based on structural connectivity is a feasible method for simulating whole-brain signals to elucidate the brain’s functional activities and network mechanisms. In order to construct a WBNM, each brain region can be represented by an NMM, and interconnections between brain regions can be constructed through excitatory connections of pyramidal neuronal populations. The functional activities of the WBNM depend on the kinetic behavior of each NMM and the interactions between multiple brain regions.

### 2.1. Coupling Strength Matrix of the WBNM

To construct a resting-state WBNM, we needed to obtain a coupling strength matrix (CSM) to simulate the interconnection of the brain regions. Usually, an empirical structural connectivity (SC) matrix can be used as a relative coupling strength matrix. An empirical resting-state functional connectivity (rsFC) matrix was used to fit the simulated rsFC of the WBNM. We selected 15 participants aged between 18 and 31, including 8 females. Participants who self-reported a history of neurological, cognitive, or psychiatric disorders were excluded from the experiment. We collected diffusion-weighted MRI, T1-weighted MRI, and fMRI data. The research was performed in compliance with the Code of Ethics of the World Medical Association (Declaration of Helsinki). Written informed consent was provided by all subjects with an understanding of the study prior to data collection, and the experiment was approved by the local ethics committee in accordance with the institutional guidelines at Charité Hospital Berlin. The human data utilized in this study originated from the preliminary research conducted by Schirner et al. [25], and they were automatically processed using the pipeline for functional MRI (fMRI) and diffusion MRI (dMRI) data. The T1-weighted MRI (TR: 1900 ms, TE: 2.52 ms, TI: 900 ms, FA: 9°, FOV: 256 mm × 256 mm × 192 mm, matrix: 256 × 256 × 192, isotropic voxel resolution: 1.0 mm), diffusion-weighted MRI (TR: 7500 ms, TE: 86 ms, FOV: 192 mm × 192 mm, matrix: 96 × 96, slices: 61, isotropic voxel resolution: 2.3 mm, diffusion directions: 64) and functional MRI (TR: 1940 ms, TE: 30 ms, FA: 78°, FOV: 192 mm × 192 mm, voxel resolution: 3 mm × 3 mm, slice thickness: 3 mm, matrix: 64 × 64, slices: 33, echo spacing: 0.51 ms, TRs: 668, eyes-closed resting state) data were acquired on a 3 Tesla Trio MRI scanner (Siemens, Berlin, Germany). Detailed insights into diffusion-weighted imaging and fMRI preprocessing can be found in the study of Schirner et al. [32]. In essence, the study integrated probabilistic white-matter fiber tractography with trajectory clustering within an automated framework and introduced a novel metric for connectivity. This metric weighted the connections between brain regions according to the minimum interface area of gray/white matter, moving away from traditional methods that rely on unweighted tract counts, which are susceptible to anatomical variability. Schirner highlighted that this approach reduces bias and yields a more accurate depiction of brain connectivity. In this study, structural connectivity matrices were extracted to represent the relative strengths of interactions between regions as mediated by white-matter fiber tracts. Empirical rsFC matrices were extracted from the BOLD signals of fMRI data.

**Figure 1:**
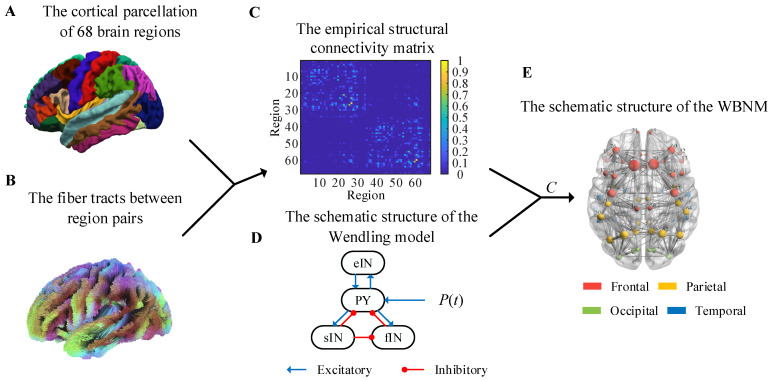
Anatomical brain network data and schematic diagram of the whole-brain network model. (**A**) The cortical parcellation of 68 brain regions. (**B**) The fiber tracts between region pairs. (**C**) The empirical structural connectivity matrix. (**D**) The schematic structure of the Wendling model. (**E**) The schematic structure of the WBNM.

T1-weighted MRIs were segmented and parcellated into 68 regions according to the Desikan–Killiany brain atlas by using FreeSurfer7.2.0 [33,34]. The cortical parcellation is shown in Figure 1A. Table 1 lists the 68 Desikan–Killiany atlas region labels, which are abbreviations of the brain region name, and provides mappings from the individual region to each lobe. The lobes were divided into the frontal, parietal, occipital, and temporal lobes. The Desikan–Killiany atlas comprised 34 cortical regions in each hemisphere. The left hemisphere’s brain regions were sequentially numbered from 1 to 34 based on the sequence number of the 34 brain regions, and the prefix LH was added before the labels. The right hemisphere’s brain regions were sequentially numbered from 35 to 68 based on the sequence number of the 34 brain regions, with the prefix RH being added before the labels.

The fiber tracts between region pairs are shown in Figure 1B, and they were obtained by applying probabilistic white-matter tractography algorithms to diffusion-weighted MRI. Then, the structural connectivity matrix of each subject could be calculated, and it was defined as the number of fibers within each region pair divided by the maximum number of fibers in all region pairs. In order to eliminate individual differences, we averaged the structural connectivity matrices of all subjects to obtain a group-representative structural connectivity matrix with the elements, *i*,*j* = 1,…68, as shown in Figure 1C. This empirical structural matrix was used as the relative coupling strength matrix to couple the NMMs to construct the WBNM. As shown in Figure 1C, the strength of the edge connections within the same hemisphere was stronger, while the strength of edge connections between hemispheres was weaker.

Then, we computed the empirical resting-state functional connectivity matrix. From functional MRI, we obtained blood-oxygen-level-dependent (BOLD) signals based on hemodynamics. The BOLD signals had a high spatial resolution with a sampling interval of 1.94 s. The first seven images of each scanning run were discarded so that the BOLD signals could reach a steady state. The representative time series in each region were extracted by averaging the time series of the voxels therein. The parcellation mask was defined according to the Desikan–Killiany atlas. We used the phase-locking value (PLV) to calculate the empirical functional connectivity matrix for each subject. Then, a group-representative empirical rsFC matrix was obtained by averaging the rsFC matrixes of all subjects.

The PLV can quantify phase coherence between two different brain regions. Firstly, the phase *θ*(*t*) was extracted with the Hilbert transform of each brain region’s time series. Then, we calculated the PLV between brain region *i* and brain region *j* with the following formula:

(1) $$\rho_{ij}=\frac{1}{T_{s}}\left|\sum_{t=1}^{T_{s}}e^{i\left[\theta_{i}(t)-\theta_{j}(t)\right]}\right|$$

where *T_s_* is the number of sample time points. The range of *ρ_ij_* is between 0 and 1. If the phase difference Δ*θ_ij_*(*t*) = *θ_i_*(*t*) − *θ_j_*(*t*) is constant over a given time window, *ρ_ij_* is equal to 1, which indicates strong phase synchronization between the two time series, whereas if the phase differences are uniformly distributed, *ρ_ij_* is approximately 0, which indicates that there is almost no phase synchronization.

In a whole-brain model, setting the coupling strength matrix between different regions of the model is a crucial and thought-provoking issue. In fact, the empirical structural connectivity matrix only provides the relative coupling strength between regions. In order to determine the value of the coupling strength matrix in the simulation model and make the functional connections of the simulation signal closer to those of an actual brain system, we need a global coupling coefficient *C* to regulate the overall strength of the long-range coupling between the regions of the WBNM:

(2) $$CS_{ij}=C\cdot C_{ij}\;\;\;i,j=1,\;2,\ldots ,68$$

where *CS_ij_* represents the elements of the coupling strength matrix of the WBNM, and *C_ij_* represents the elements of the empirical structural connectivity matrix.

In the study, we needed to select an appropriate parameter value for the global coupling coefficient *C* to achieve the highest correlation between the simulated rsFC matrix and the empirical rsFC matrix. We used the PLV to calculate the simulated rsFC matrix with different global coupling coefficients *C* and the empirical rsFC matrix for 68 brain regions. Then, by calculating the Pearson correlation coefficient (PCC) between the elements in the lower triangle of the simulated rsFC matrix and the corresponding positions in the empirical rsFC matrix, a similarity measure of functional connections between the simulated brain network and the empirical brain network was obtained in different situations of the global coupling coefficient *C*. The *C* value corresponding to the strongest similarity was the most suitable point, and we could obtain the most suitable coupling strength matrix for the WBNM.

### 2.2. The Nodes of the WBNM

The selection of nodes for a WBNM is also important. Figure 1D shows a diagram of the Wendling model’s structure. The Wendling model was used to construct each brain region in the WBNM due to its rich dynamic characteristics and relatively simple structure. The Wendling model comprises four main components: excitatory pyramidal neurons, fast inhibitory interneurons, slow inhibitory interneurons, and background activity. The excitatory neurons are responsible for transmitting excitatory signals, while the fast and slow inhibitory neurons provide short-term and long-term network inhibition, respectively [18]. These components are interconnected through mathematical equations that simulate the dynamic interactions between neurons and how these interactions collectively influence the brain’s electrical activity patterns. Background activity comprises external inputs and internal noise, adding randomness and complexity to the model [18]. The dynamic characteristics of each node also affect the entire brain network. Figure 1E illustrates the architectural framework of the WBNM. The left part of Figure 1E uses different-colored spheres to represent the distribution of the 68 brain regions, with red representing frontal, yellow representing parietal, green representing occidental, and blue representing temporary. The size of each sphere represents the strength of the node, indicating its degree of synchronization with other nodes in the network. Further details on this will be discussed later in Section 2.3.

**Figure 2:**
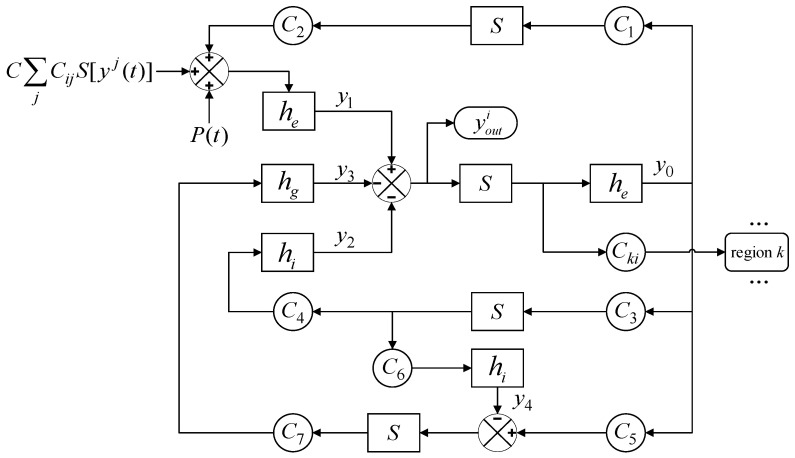
A block diagram of the whole-brain network model.

A block diagram of the Wendling model with mutual coupling between different regions is shown in Figure 2. Within the Wendling model, the transformation functions, which are denoted as *he*, *hi*, and *hg* for excitatory, slow inhibitory, and fast inhibitory post-synaptic membrane potentials, respectively, are defined through mathematical formulations. These functions effectively transduce the average pulse density of action potentials into corresponding synaptic potentials, offering a quantitative framework for capturing the nuanced dynamics of glutamatergic excitation, GABA_A-mediated fast inhibition, and GABA_B-mediated slow inhibition within neural networks. *S* delineates the nonlinear characteristics of neuronal excitability thresholds, mapping input signals to a bounded output range to simulate the activation mechanisms of neurons and their saturation features. *C* represents a global coupling coefficient that can rescale the structural connectivity strength between regions. *C_ij_* represents the structural connectivity strength from region *j* to region *i*. $y_{out}^{i}$ represents the output of brain region *i* and the input to other brain regions. The external inputs from the surrounding environment or external disturbances are encapsulated by the term *P*(*t*). These inputs are mathematically modeled as Gaussian white noise, which is characterized by a mean (*μ*) and variance (*σ*^2^).

The linear dynamic transform functions play a crucial role in converting the average pulse density of action potentials into post-synaptic membrane potentials, which cover both excitatory and inhibitory variations:

(3) $$h(t)=\left\{\begin{array}{ll} Wwte^{-wt} & ,t\geq 0\\ 0 & ,t<0 \end{array}\right.$$

where *W* ∈ {*A*, *B*, *G*}, *w* ∈ {*a*, *b*, *g*}, *W* denotes the average synaptic gains, and *w* symbolizes the inverse of the average time constants.

The impulse response in Formula (3) corresponds to the following two first-order linear ordinary differential equations:

(4) $$\left\{\begin{array}{l} \dot{y}(t)=z(t)\\ \dot{z}(t)=Wwx(t)-2wz(t)-w^{2}y(t) \end{array}\right.$$

where *x*(*t*) represents the input average pulse density of the action potential, and *y*(*t*) represents the convolution output’s post-synaptic membrane potential.

The *S* function is a crucial component that describes the response of the average membrane potential to post-synaptic currents. This function is nonlinear, is typically represented as a sigmoid function, and is given by

(5) $$S(v)=\frac{2e_{0}}{1+e^{r(v_{0}-v)}}$$

where 2*e*_0_ represents the maximum firing rate, i.e., the maximum output frequency of the neuron, *v* is the average membrane potential, *v*_0_ is the half-activation potential, meaning that when *v*_0_ = *v*, the output frequency is *e*_0_, and *r* is a slope parameter that determines the shape of the *S* function.

In summary, node *i* of the WBNM can be described by the following first-order differential equations:

(6) $$\begin{array}{l} {\dot{y}}_{0}^{i}(t)=y_{5}^{i}(t)\\ {\dot{y}}_{1}^{i}(t)=y_{6}^{i}(t)\\ {\dot{y}}_{2}^{i}(t)=y_{7}^{i}(t)\\ {\dot{y}}_{3}^{i}(t)=y_{8}^{i}(t)\\ {\dot{y}}_{4}^{i}(t)=y_{9}^{i}(t)\\ {\dot{y}}_{5}^{i}(t)=AaS[y_{1}^{i}(t)-y_{2}^{i}(t)-y_{3}^{i}(t)]-2ay_{5}^{i}(t)-a^{2}y_{0}^{i}(t)\\ {\dot{y}}_{6}^{i}(t)=Aa\{p(t)+C\sum_{j}C_{ij}S[y^{j}(t)]+C_{2}S[C_{1}y_{0}^{i}(t)]\}-2ay_{6}^{i}(t)-a^{2}y_{1}^{i}(t)\\ {\dot{y}}_{7}^{i}(t)=BbC_{4}S[C_{3}y_{0}^{i}(t)]-2by_{7}^{i}(t)-b^{2}y_{2}^{i}(t)\\ {\dot{y}}_{8}^{i}(t)=GgC_{7}S[C_{5}y_{0}^{i}(t)-y_{4}^{i}(t)]-2gy_{8}^{i}(t)-g^{2}y_{3}^{i}(t)\\ {\dot{y}}_{9}^{i}(t)=BbC_{6}S[C_{3}y_{0}^{i}(t)]-2by_{9}(t)-b^{2}y_{4}(t) \end{array}$$

where $y_{0}^{i}$ to $y_{9}^{i}$ are the state variables of region *i*, *i* = 1,2,…68. *y^j^*, *j* = 1,2,…68 is the output of region *j*, which couples with region *i*.

The output of region *i* is the post-synaptic potential of the pyramidal cell population, which is defined as

(7) $$y_{out}^{i}(t)=y_{1}^{i}(t)-y_{2}^{i}(t)-y_{3}^{i}(t)$$

The Wendling model was employed for all brain regions with identical parameter values. The standard numerical values and their physiological interpretations are listed in Table 2, as referenced in [18].

In this study, the WBNM was solved with the fourth order Runge-Kutta algorithm in Matlab 2018b. The sampling rate was set to 1000 Hz, and the simulation length was set to 2 s. We discarded the first 1 s of the simulated signals for each brain region to eliminate the influence of the initial values. Simulations with different parameters were repeated 20 times with different initial values.

### 2.3. Methods of Analyzing the Brain Functional Network Based on Graph Theory

Graph theory analysis plays a pivotal role in understanding the functional connectivity networks of the brain. By conceptualizing the brain as a complex network at a macroscopic level, with nodes representing brain regions and edges representing connection strengths, the construction of adjacency matrices for graph-theoretical metrics allows us to precisely quantify and characterize the connectivity patterns between different regions of the brain. In this study, graph theory was used to obtain indicators for measuring the network characteristics at the node, edge, local network, and global network levels.

Before calculating the network metrics in graph theory, we first constructed an adjacency matrix using the absolute thresholding method. By setting a threshold, all values in both the simulated rsFC matrix and the empirical rsFC matrix were compared with this threshold. Elements greater than or equal to the threshold were considered as edges in the network (connections exist), while those less than the threshold were regarded as non-connected. The range of threshold values explored was from 0 to 0.5, with a step size of 0.01, resulting in a total of 51 thresholds. The network’s graph theory properties were calculated at each threshold value.

The most commonly used measures in graph theory for analyzing complex brain networks are the node strength, node degree, characteristic path length, clustering coefficient, and global efficiency. These metrics comprehensively reflect a network’s topological characteristics from various perspectives, including but not limited to those of information transmission efficiency, clustering properties, and key brain regions. They are used to compare the structural and functional similarities and differences between a WBNM and empirical brain networks. These measures were calculated by using the Brain Connectivity Toolbox in this study. In the following, we provide an introduction to these five metrics.

(1) Node strength: Strength represents a fundamental metric for quantifying the connectivity and importance of nodes within weighted networks. The calculation of the strength of node *i* is defined as

(8) $$S_{i}=\sum_{j\in N}{C^{\prime }}_{ij}$$

where ${C^{\prime }}_{ij}$ represents the weight of the edge connection between node *i* and node *j*, and *N* denotes the set of nodes that are adjacent to node *i*. Larger node strengths point to central influential positions with critical roles in maintaining network connectivity and function. In contrast, smaller node strengths signify peripheral positions with less influence on the network’s global structure.

(2) Node degree: This degree measures the number of edges that a node shares with other nodes. The calculation of the degree of node *i* is defined as

(9) $$D_{i}=\sum_{j\in N}a_{ij}$$

where $a_{ij}\in \left\{0,1\right\}$ represents whether there is an edge connection between node *i* and node *j*. The degree is the most basic network metric, and most other metrics are related to the node degree. The smaller the value of *D_i_*, the smaller the role of node *i* in network information transmission. The larger the value of *D_i_*, the greater the role of node *i* in network information transmission.

The average degree of a network is a key metric in network analysis that quantifies the typical level of connectivity of nodes within a network. It is calculated as the mean value of the degrees of all nodes in the network, providing a simple yet insightful measure of the network’s overall connectivity and density. The calculation of the average degree of a network is defined as

(10) $$D=\frac{1}{N}\sum_{i=1}^{N}D_{i}$$

where *N* represents the total number of nodes in the network. *D* = 1 indicates that there are edge connections between any two nodes in the network, while *D* = 0 indicates that all nodes in the network are isolated nodes without edge connections.

(3) Characteristic path length: The characteristic path length is generally defined as the average of the shortest path lengths between all possible pairs of nodes in a network. The calculation of the characteristic path length of a network is defined as

(11) $$L=\frac{1}{N(N-1)}\sum_{i,j\in N,i\neq j}d_{ij}$$

where *N* represents the total number of nodes in the network, and *d_ij_* represents the shortest path length between node *i* and node *j*. The maximum number of edges that may exist in an undirected network is *N*(*N* − 1)/2. The value of the characteristic path length, *L*, is inversely related to the speed of information transmission within a network. Specifically, a smaller value of *L* signifies that the network facilitates quicker information exchange. Conversely, a larger value of *L* suggests a slower information transmission speed.

(4) Clustering coefficient: The clustering coefficient for a node *i* describes the ratio of the actual number of edges *e_i_* between the neighbors of *i* to the maximum possible number of edges between them. For node *i*, the local clustering coefficient *CC_i_* is defined as

(12) $$CC_{i}=\frac{2e_{i}}{k_{i}(k_{i}-1)}$$

where *e_i_* represents the actual number of edges between node *i* and its neighboring nodes, and *k_i_* is the degree of node *i*, which indicates the number of neighbors. The range of variation in *CC_i_* is between 0 and 1. The smaller the value of *CC_i_*, the more dispersed the nodes are and the sparser the network is. The larger the value of *CC_i_*, the more clustered the nodes are and the closer the network is.

The global clustering coefficient can be understood as the average of the local clustering coefficients of all nodes in the network, and it provides a measure of the network’s overall tendency to cluster. Thus, the global clustering coefficient *CC* can also be calculated through the following formula:

(13) $$CC=\frac{1}{N}\sum_{i=1}^{N}CC_{i}$$

where *N* represents the total number of nodes in the network, and *CC_i_* is the local clustering coefficient of node *i*. When *CC* = 1, this indicates that any two nodes in the network are adjacent nodes. When *CC* = 0, this indicates that there are no adjacent nodes between all nodes in the network.

(5) Global efficiency: The global efficiency is a metric used in network analysis to assess the overall efficiency of information transfer within a network. It is based on the shortest path lengths between pairs of nodes in the network, and it reflects the network’s ability to process and transmit information on a global scale. The formula for calculating the global efficiency is defined as the average efficiency between all pairs of nodes in the network, and it is mathematically expressed as

(14) $$E=\frac{1}{N(N-1)}\sum_{i,j\in N,i\neq j}\frac{1}{d_{ij}}$$

where *N* represents the total number of nodes in the network, and *d_ij_* represents the shortest path length between node *i* and node *j*. The range of variation in *E* is between 0 and 1. The smaller the value of *E*, the lower the efficiency of network information exchange. The higher the value of *E*, the higher the efficiency of network information exchange.

The small-world network index can be used measure whether a network is a small-world network that lies somewhere between a regular network and a random network. Small-world networks have the characteristics of high clustering coefficients and low characteristic path lengths. The calculation of the small-world network index of a network is defined as follows:

(15) $$\sigma =\frac{\gamma }{\lambda }=\frac{CC_{real}/CC_{random}}{L_{real}/L_{random}}$$

where *γ* represents the clustering coefficient ratio, and *λ* represents the characteristic path length ratio. *CC_real_* and *L_real_* represent the clustering coefficients and characteristic path lengths of the network to be analyzed, respectively. *CC_random_* and *L_random_* represent the clustering coefficients and characteristic path lengths of the random network corresponding to the network to be analyzed, respectively.

## 3. Results

### 3.1. The Fitting of the Simulated rsFC Matrix and the Empirical rsFC Matrix

**Figure 3:**
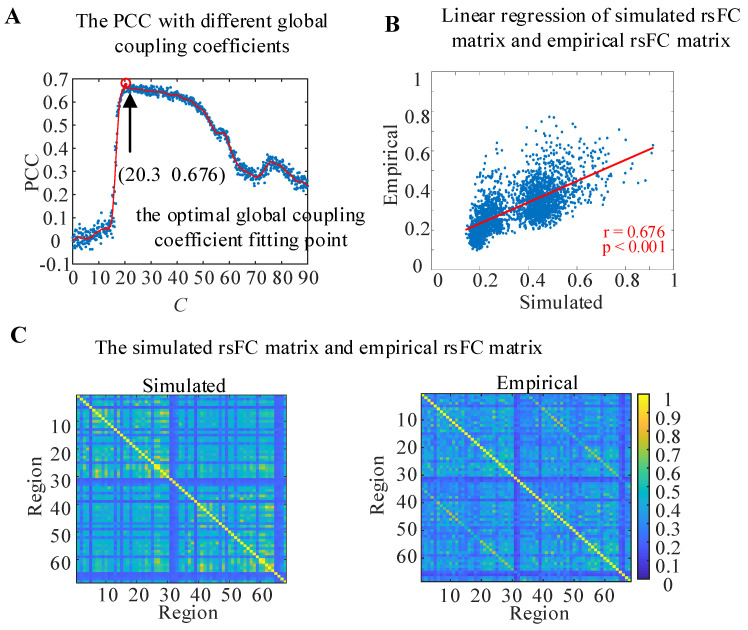
The WBNM at the optimal global coupling coefficient fitting point. (**A**) The PCC between the simulated rsFC matrix and empirical rsFC matrix with different global coupling coefficients. (**B**) Linear regression of the simulated rsFC matrix and empirical rsFC matrix. (**C**) The simulated rsFC matrix and empirical rsFC matrix.

In order to make the functional connections of the WBNM closer to the empirical connections of an actual brain, the Pearson correlation coefficient (PCC) between the simulated rsFC matrix of the WBNM and the empirical rsFC matrix with different global coupling coefficients *C* and with a step size of 0.1 was calculated and is shown in Figure 3A. The scatter points were marked in blue, and a red solid line was used to fit the scatter plot to represent the trend of correlation changes with different global coupling coefficients, for which the Savitzky–Golay filtering method was adopted. The larger the correlation value, the better the simulated brain functional connection fit the empirical brain functional connection. The results showed that when the global coupling coefficient increased, the correlation showed a trend of first increasing and then decreasing. The correlation increased when *C* was greater than 70 and then decreased again when *C* was greater than 75. The correlation reached a maximum value of 0.676 with a significance level of *p* < 0.001 when the global coupling coefficient was *C* = 20.3, as marked with a red circle. At this point, the simulated rsFC matrix of the constructed WBNM and the empirical rsFC matrix had the highest similarity, and the WBNM was closest to an actual brain network. Therefore, all subsequent analyses of the WBNM were based on the global coupling coefficient *C* = 20.3, which is called the optimal global coupling coefficient fitting point.

To observe the linear correlation between the simulated and empirical rsFC matrixes at the optimal global coupling coefficient fitting point in detail, a linear regression was calculated, and the results are shown in Figure 3B. The horizontal axis represents the values of the lower triangular elements of the simulated rsFC matrix, while the vertical axis represents the values of the lower triangular elements of the empirical rsFC matrix at the same position. The red line represents the linear regression fitting results of the scatter plot. The PCC between the simulated rsFC matrix and the empirical rsFC matrix at the optimal global coupling coefficient fitting point reached 0.676 and *p* < 0.001.

The details of the simulated rsFC matrix and the empirical rsFC matrix are shown in Figure 3C. The values of the simulated rsFC matrix and the empirical rsFC matrix were similar in both the intra-hemispheric and inter-hemispheric regions, while in most brain regions, the values of the simulated rsFC matrix were higher than those of the empirical rsFC matrix.

### 3.2. The Characteristics of the Output Signals of the WBNM

**Figure 4:**
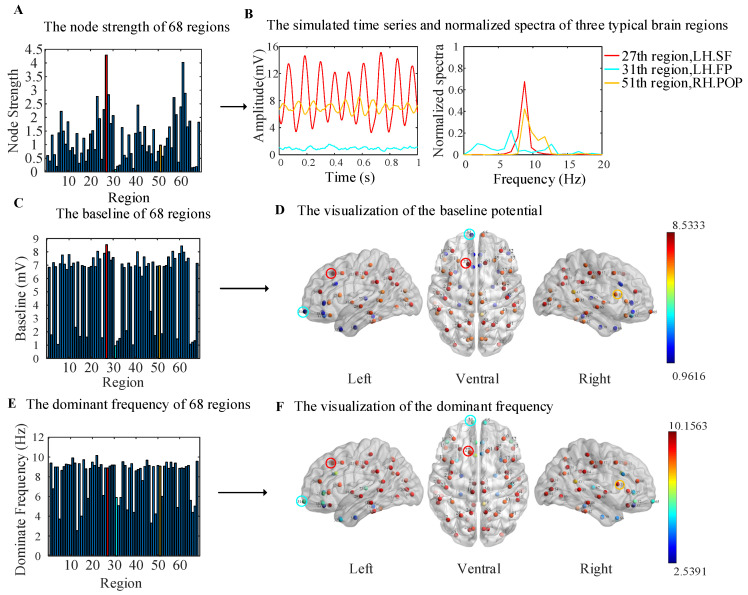
The dynamics of the simulated output signals for all regions. (**A**) A bar plot of the node strength. (**B**) The simulated time series and normalized spectra of 27, 31, and 51 brain regions. (**C**) A bar plot of the baseline potential for all brain regions. (**D**) A visualization of the baseline potential from the left, ventral, and right angles of view. (**E**) A bar plot of the dominant frequencies for all brain regions. (**F**) A visualization of the dominant frequencies from the left, ventral, and right angles of view.

In the WBNM, each node represented a brain region. In order to analyze the dynamics of the simulated output signals of each brain region more clearly, we first calculated the node strength of each brain region. Figure 4A shows the node strengths of each brain node, which were basically symmetrical in the left and right hemispheres. The highest node strength corresponded to the 27th brain region (LH.SF), which is marked with red color, and the lowest node strength corresponded to the 31th brain region (LH.FP), which is marked with bright blue. The 51th brain region (RH. POP) showed a moderate node strength, which is marked with orange. The stronger the node strength, the greater the importance of information transmission in the brain network; such nodes are commonly referred to as hub nodes. The smaller the node strength, the less important it is for information transmission in the brain network.

Displaying the simulated time series and corresponding normalized spectra of all 68 brain regions would make the figure very chaotic, so we selected three representative brain regions, as shown in Figure 4B. The simulated output signal of brain region 27 (LH. SF), which had the highest node strength, showed high-amplitude, high-frequency oscillations, with a peak frequency that fell on the alpha band at about 8 Hz; this is marked in red. The output of brain region 31 (LH. FP), which had the lowest node strength, showed characteristics of EEG background noise with a low amplitude and a broadband spectrum; this is marked in bright blue. It can be seen that the coupling strength between the region 31 and other regions was very low in both the simulated rsFC matrix and empirical rsFC matrix in Figure 3C. Therefore, region 31 showed the characteristics of EEG background noise. The output of brain region 51 (RH. POP), which had moderate node strength, is marked in orange. It also showed alpha-wave oscillation, with a lower amplitude and slightly wider spectrum than those of region 27; the dominant frequency was also about 8 Hz.

The baseline potential and dominant frequency of the simulated output signals for all regions are shown in Figure 4C–F. In Figure 4C, the bar plot shows the baseline potential, which was visualized on left, ventral, and right cortical surface MNI templates in Figure 4D by using the BrainNet Viewer toolbox [35]. The color of the sphere represents the magnitude of the baseline potential, and the three representative brain regions are marked with red, bright blue, and orange circles. The baseline potentials of the corresponding brain regions in the left and right hemispheres were basically symmetrical. The brain regions with a baseline of larger than 4 mV were almost located in the parietal and occipital regions and are marked with red color, and those with less than 4 mV were located in the frontal and temporal regions and are marked with blue color, as shown in Figure 4D.

In Figure 4E, the bar plot shows the dominant frequency, and a visualization is shown in Figure 4F. The dominant frequencies of the corresponding brain regions in the left and right hemispheres were also basically symmetrical. The brain regions with an alpha dominant frequency (8–12 Hz) were consistent with those with a baseline frequency above 4 mV in the parietal and occipital regions. The values for the other brain regions were less than 8 Hz, and they are marked with blue color in the frontal and temporal regions.

Comparing Figure 4A,C,E, it can be seen that the brain regions with lower baseline potential values and dominant frequencies corresponded to lower node strength values, indicating that these regions had lower participation in network communication, thus representing a background EEG state.

### 3.3. The Characteristics of the Brain Network Based on Graph Theory

A brain network can be represented as a graph of nodes connected to each other through edges, where nodes represent brain regions and edges represent the strength of functional connections between them. Usually, elements with a functional connection value between nodes greater than the threshold are set to 1, and the elements with a value less than the threshold are set to 0. Then, the constructed adjacency matrix can be used to analyze the graph-theoretical measures of brain networks.

**Figure 5:**
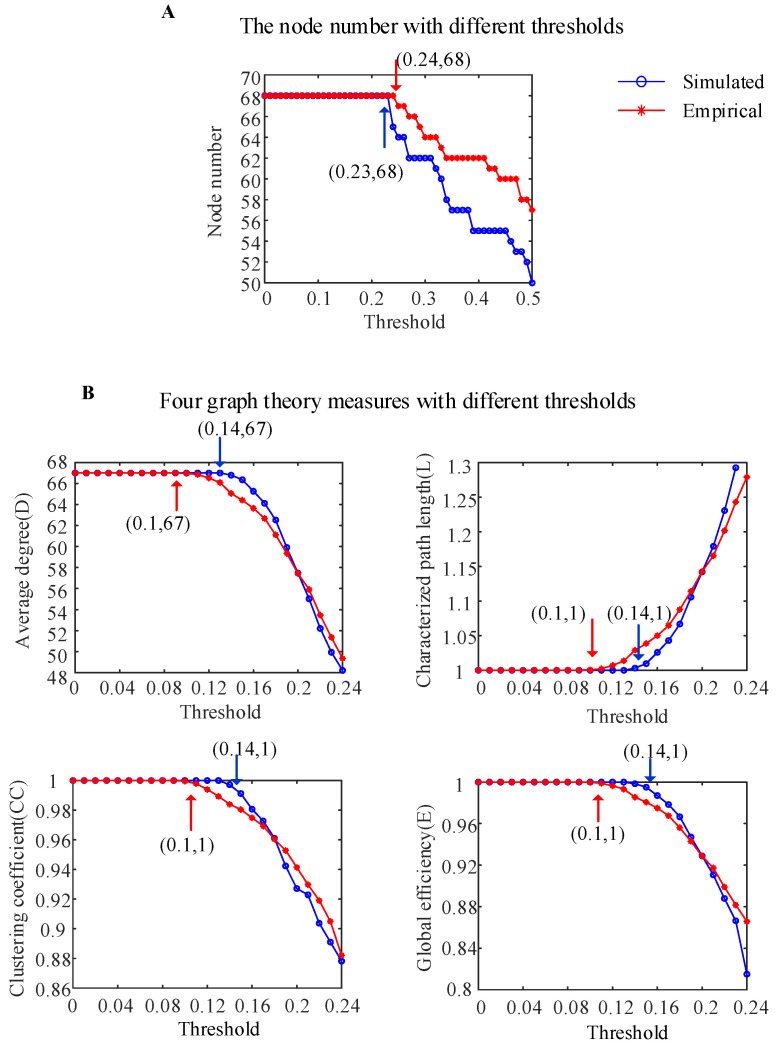
The characteristics of the simulated WBNM and empirical brain network based on graph theory. (**A**) The node numbers with different thresholds for the two networks. (**B**) Four graph-theoretical measures with different thresholds for the two networks.

Figure 5A shows the variation curves of the numbers of nodes in the simulated WBNM and empirical brain networks under different thresholds (the calculation details of the adjacency matrix are provided in the Section 2). The blue solid line represents the variation curve of the number of nodes in the simulated brain network, while the red solid line represents the empirical brain network. Simulated brain networks and empirical brain networks exhibit isolated nodes without edge connections when the thresholds are greater than 0.23 and 0.24, respectively. Therefore, we chose to observe the four commonly used graph-theoretical measures with thresholds ranging from 0 to 0.24.

To investigate the similarity of the brain network characteristics between the simulated WBNM and empirical brain networks, the four commonly used graph-theoretical measures were calculated under different thresholds, as shown in Figure 5B (the calculation details of the graph theory measures are provided in the Section 2). When the threshold was less than 0.14 for the simulated WBNM and less than 0.1 for the empirical brain network, as marked with the blue and red arrows, respectively, the average degree *D* was equal to 67, indicating that all nodes in the brain network had edge connections. In the same case, the characteristic path lengths *L* were equal to 1, which indicated that the shortest path lengths between all nodes in the brain network were one edge, the clustering coefficients *CC* were equal to 1—indicating that nodes in the network were highly clustered—and the clustering coefficients *E* were equal to 1—indicating that the information transmission efficiencies between the brain network nodes were the highest. From the analysis of the four graph-theoretical measures, including the average degree, characteristic path length, clustering coefficient, and global efficiency, it was evident that the WBNM and empirical brain networks exhibited strong similarities in their network characteristics. However, at certain thresholds, the simulated network demonstrated a slightly higher efficiency in information transmission compared to that of the empirical network. Beyond a certain threshold, this relationship was reversed.

### 3.4. Small-World Network Properties

Watts and Strogatz [36] proposed the concept of small-world networks in 1998. Small-world networks lie somewhere between regular networks and random networks, and they have the characteristics of a high clustering coefficient and a low characteristic path length. Furthermore, Liao et al. [37] demonstrated that complex human brain networks also have small-world network properties.

**Figure 6:**
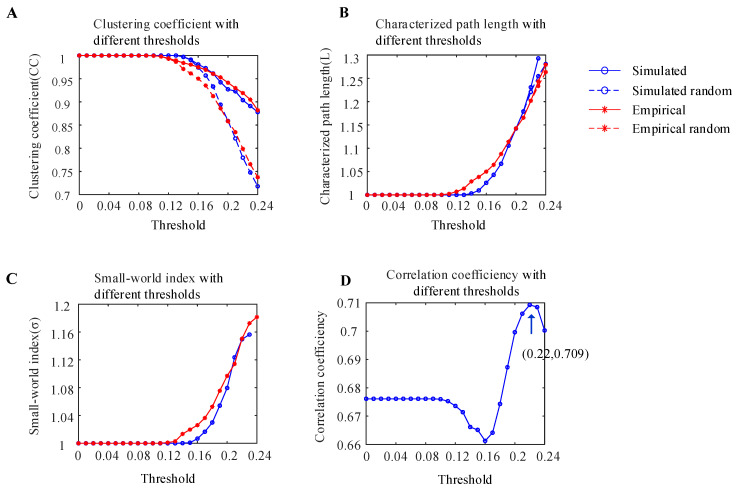
Analysis of the small-world characteristics of the simulated WBNM and empirical brain network. (**A**) The clustering coefficients of the simulated WBNM, simulated random WBNM, empirical brain network, and empirical random brain network with different thresholds. (**B**) The characteristic path lengths of the simulated WBNM, simulated random WBNM, empirical brain network, and empirical random brain network with different thresholds. (**C**) The small-world network indexes of the simulated WBNM and empirical brain network with different thresholds. (**D**) The correlation of the functional connectivity matrix between the simulated WBNM and empirical networks with different thresholds.

To measure whether the simulated WBNM and empirical brain network constructed in this experiment had small-world network properties, the clustering coefficients and characteristic path lengths of simulated random brain networks and empirical random brain networks with the same numbers of nodes and edges were calculated under different thresholds, as shown in Figure 6A,B. As the threshold increased, the clustering coefficients *CC* of all four types of brain networks showed a downward trend, and the clustering coefficients *CC* of the simulated and empirical brain networks were greater than those of random brain networks. The characteristic path lengths *L* of the four types of brain networks showed an upward trend, and the characteristic path lengths *L* of the simulated and empirical brain networks were similar to those of random brain networks.

The small-world network index is expressed as the ratio of the clustering coefficient ratio divided by the characteristic path length ratio, and it is used to determine whether a network has small-world properties. A small-world network index σ that is greater than 1 indicates that the network has small-world properties. The small-world network indices of the simulated WBNM and empirical brain networks with different thresholds are shown in Figure 6C. As the threshold increased, both small-world network indices showed an upward trend and were greater than 1, which indicated that both the simulated WBNM and empirical network had small-world network properties.

The Pearson correlation coefficients of the functional connectivity matrix between the simulated WBNM and empirical networks with different thresholds are shown in Figure 6D. As the threshold increased, the correlation first decreased and then increased. At the threshold of 0.22, the correlation achieved its maximum value of 0.709, indicating a high degree of similarity between the functional connectivity matrixes of the simulated WBNM and empirical networks at this threshold.

## 4. Discussion

The purpose of this study was to construct a whole-brain network that was close to an actual brain in terms of EEG rhythm, functional connectivity, and brain network characteristics. In this way, researchers can use this whole-brain model to study the neural mechanisms of EEG signal generation, the pathogenic mechanisms of various functional diseases of the brain, and various methods of brain stimulation regulation from a multi-scale perspective. Using computational models of brain dynamics to construct a whole-brain model is not an unprecedented effort. In fact, our work was inspired by previous large-scale modeling studies, which elucidated key insights into using various other models to construct an entire brain network. The innovation of this study lies in its introduction of a Wendling model with richer dynamic characteristics to represent the nodes of the brain network, and at the same time, real MRI data and an optimized global coupling coefficient were used to simulate the connections of the brain network, thus constructing a WBNM that was closer to the real brain in terms of brain rhythm, functional connectivity, and network characteristics.

In this study, we successfully constructed a comprehensive brain model to simulate a real whole-brain EEG network, and it was able to balance physiological accuracy and computational processability. Firstly, we partitioned the entire brain based on the Desikan–Killiany brain atlas and then calculated the quantity of white-matter fiber bundles found between different brain regions, which enabled us to derive a relative coupling strength matrix for whole-brain coupling. By coupling 68 Wendling NMMs and optimizing a global coupling coefficient of *C* = 20.3, we achieved the optimal simulation for the functional connectivity matrix of a real brain network. Then, we analyzed the dynamics of the simulated EEG and employed graph-theoretical metrics to further characterize the attributes of the constructed WBNM, namely, the average degree, characteristic path length, clustering coefficient, global efficiency, and small-world properties. We found that the EEG spectra and network properties of the WBNM constructed in this study were highly similar to those of a real brain network.

In this study, the Wendling model was selected as the node of the whole-brain network, and it made an important contribution to the development of the WBNM. To the best of our knowledge, the utilization of the Wendling model for computational modeling at the whole-brain scale has not been previously explored. When simulating a neural system from a microscopic perspective, it is usually necessary to model a large number of spike discharges of neuronal membrane voltage, which can cause significant computational complexity. It is also difficult to determine how a large number of neurons connect to each other. Therefore, some studies chose to use the “mean field theory” neural mass model as the node of the whole-brain network. An NMM uses a set of differential equations to represent the coupled excitatory and inhibitory neuronal populations, which can represent the true physiological meaning of neuronal populations, such as the population firing rate and postsynaptic current. However, in most previous studies and research involving whole-brain problems, in order to reduce the complexity of WBNMs, the selected models of individual neural units were highly idealized, and the structure of neural group units was overly simplified, as they usually consisted of basic excitatory and inhibitory loops, such as in the Wilson–Cowen model, Later Breakspeed model, Arm model, and Jansen model [1,2,24,25,26,28]. As a result, the whole-brain models that they constructed had certain differences from the actual brain structure. In comparison, the Wendling model can generate six common EEG signals, including background noise, alpha waves, epileptic spike waves, etc., thus enhancing the physiological and physical reality in certain aspects. The signals simulated in the Wendling model have rich dynamic characteristics and wider frequency bands and are closer to real human EEG features [38,39,40,41]. Ursino et al. [22] proposed an expanded Wendling model to faithfully replicate actual brain EEG signals, they and obtained different rhythm combinations (β and γ, α and γ, or a wide spectrum). However, the Ursino model is somewhat complex.

Of course, the Wendling model also has some limitations of its own. Although the Wendling model can simulate EEG signals with rich dynamic characteristics, there is still a certain gap with respect to the simulation of real EEG signals because the characteristics of EEG signals are very complex. For example, the Wendling model cannot generate gamma waves and ripple waves. The Wendling model is also a model with very complex bifurcation dynamics. Improper parameter settings and mutual coupling that is too strong may cause the Wendling model to be in an abnormal state. The Wendling model currently has a wealth of research results focused on epilepsy, with relatively few studies on other functional disorders of the brain and cognition.

We selected the Wendling model as the node of the WBNM. Therefore, using the Wendling model as the node of WBNM was beneficial for reproducing a real-world brain neural network while also maintaining a balance between authenticity and computational complexity. In this study, the simulated EEG exhibited rich rhythmic characteristics, including alpha oscillations, a wider spectrum, and the background state (Figure 4). The results indicated that using the Wendling model as a node made the EEG features generated by the WBNM closer to reality. Moreover, by adjusting the parameters of the Wendling model, the dynamic states of different regions could also be adjusted, which can be beneficial when using this WBNM to conduct research on epilepsy transmission and other phenomena.

Another advantage of the WBNM constructed in this study is based on the clear anatomical structure and the use of real MRI signals to form a structural connectivity matrix. The fiber tracts that were calculated from MRI data could represent the relative coupling strength between different regions. However, during the modeling process, the value of the fiber tracts was different from the value of the coupling coefficient between the regions. To keep the relative coupling strength between regions unchanged, we introduced the global coupling coefficient C, which used a multiplicative factor to adjust the specific coupling strength between regions in the model. By calculating the correlation between the functional connectivity matrix of the WBNM and the functional connectivity matrix of the empirical data, the value of C = 20.3 at the strongest correlation point was determined as the optimal global coupling coefficient. By setting the global coupling coefficient to *C* = 20.3, the rsFC matrices of the simulated WBNM and empirical brain network had strong similarity, and the PCC value reached 0.676 (Figure 3). Therefore, the simulated brain network and empirical brain network had similar graph-theoretical measurements (Figure 5). Both of them have small-world properties, and when the threshold value was 0.22, there was a strong correlation between the simulated rsFC matrix and the empirical rsFC matrix with PCC = 0.709 (Figure 6). All of the above results indicate that the connection characteristics and network characteristics of the WBNM proposed in this study are very similar to those of actual brain networks. In some platforms that built whole-brain models [1,2], different models could be selected as the nodes of the whole-brain network (these models were relatively simple compared to Wendling models), and real structural or functional datasets could be used to form a functional coupling matrix. However, there is no evidence to prove whether the WBNMs that they constructed would generate brain-like EEG waveforms or real-world brain network features.

There are also some limitations in this study. For example, although the flexibility of the code in our constructed WBNM gave it a certain scalability, we still needed to set the global coupling coefficient *C* to make it fit an actual neural system in terms of EEG waveforms at each node, functional connections, and brain network characteristics. When other subjects’ structural connectivity data are used, it is necessary to select a new global coupling coefficient. If we want to change or add brain regions, in the initial step of modeling, which is the calculation of the fiber tracts, we need to recalculate, and the entire model construction process needs to be repeated, which is very time-consuming and laborious. However, we believe that in order to build a more realistic WBNM, this work is necessary. Building a WBNM that simulates the real human brain is not a simple task. Further optimizing the behavior of brain regions requires more complex node models or the optimization of the parameters of each node model. Due to the large number of model parameters, optimizing parameter settings at the whole-brain level is very difficult. However, we can start by reverse-identifying the model parameters of one or several brain regions in order to make the WBNM as close to the actual situation as possible. Our study only constructed a WBNM and did not use this model to study the neurophysiological mechanisms of the brain or functional diseases of the brain. The application aspect is also the direction of our future work.

In future work, our team and other researchers can use this model to perform meaningful work. For example, by adjusting the parameters of the Wendling model, including the excitability, inhibition, and synaptic connectivity coefficient, as well as the structural coupling matrix, we can investigate the mechanisms of reduced brain rhythms, complexity, and functional connectivity in AD and MCI patients. Thus, we can conduct research on the brain functional mechanisms and intervention measures in AD and MCI patients. By adjusting the parameters of the Wendling model, including the excitability, inhibition, and synaptic connectivity coefficient, as well as the structural coupling matrix, we can investigate the brain mechanisms of the reduced brain rhythm, complexity, and functional connectivity in AD and MCI patients. By adding electrical stimulation to appropriate parts of this WBNM, we can investigate the possibility of intervening in cognitive decline. For example, researchers can work on applying direct-current electrical stimulation to this WBNM to investigate the effects of different intensities and positions of electrical stimulation on the waveforms, dynamic behavior, and network characteristics of the whole-brain model’s output. Similarly, we can also simulate the spread of epilepsy in the brain and study feasible methods for the closed-loop control of seizures.

## 5. Conclusions

In this study, we constructed a resting-state whole-brain network model (WBNM) with 68 brain regions using the Wendling model for the nodes with real MRI data forming the interconnections. By fine-tuning the global coupling coefficient to optimize the correlation between the simulated rsFC matrix and the empirical rsFC matrix, the WBNM had functional connections that were similar to real EEG signals. The EEG signals of network nodes with different node degrees exhibited different rhythms, bandwidths, baseline potentials, and dominant frequencies. Then, we compared the differences between the simulated and empirical brain networks at different thresholds using four widely used graph-theoretical measures. The WBNM that we constructed had network features that were similar to those of empirical neural networks, including the average degree, characteristic path length, clustering coefficient, and global efficiency, as well as small-world network attributes. We suggest that the constructed Wendling WBNM can reproduce real brain networks to a certain extent and can be used to study the neurophysiological mechanisms of cognitive and functional disorders of the brain.

## Figures and Tables

**Table 1:** Parcellation and corresponding lobes of the Delikan–Killiany brain regions.

Number	Region	Label	Lobe	Number	Region	Label	Lobe
1	Banks of Sup. Temp. Sulcus	BK	Temporal	18	Pars orbitalis	POR	Frontal
2	Caudal anterior cingulate	CAC	Frontal	19	Pars triangularis	PTR	Frontal
3	Caudal middle frontal	CMF	Frontal	20	Pericalcarine	PCL	Occipital
4	Cuneus	CU	Occipital	21	Postcentral	POC	Parietal
5	Entorhinal	EN	Temporal	22	Posterior cingulate	PCG	Parietal
6	Fusiform	FU	Temporal	23	Precentral	PRC	Frontal
7	Inferior parietal	IP	Parietal	24	Precuneus	PCU	Parietal
8	Inferior temporal	IT	Temporal	25	Rostral anterior cingulate	RAC	Frontal
9	Isthmus cingulate	IST	Parietal	26	Rostral middle frontal	RMF	Frontal
10	Lateral occipital	LO	Occipital	27	Superior frontal	SF	Frontal
11	Lateral orbitofrontal	LOF	Frontal	28	Superior parietal	SP	Parietal
12	Lingual	LG	Occipital	29	Superior temporal	ST	Temporal
13	Medial orbitofrontal	MOF	Frontal	30	Supramarginal	SMG	Parietal
14	Middle temporal	MT	Temporal	31	Frontal pole	FP	Frontal
15	Parahippocampal	PH	Temporal	32	Temporal pole	TP	Temporal
16	Paracentral	PAC	Frontal	33	Transverse temporal	TT	Temporal
17	Pars opercularis	POP	Frontal	34	Insula	IN	Parietal

**Table 2:** Standard values and physiological interpretations of the parameters in the Wendling model.

Parameter	Interpretation	Standard Value
A	Average excitatory synaptic gain	3.25 mV
B	Average slow inhibitory synaptic gain	22 mV
G	Average fast inhibitory synaptic gain	10 mV
a	Reciprocal of the average excitatory time constant	$100\;s^{-1}$
b	Reciprocal of the average slow inhibitory time constant	$50\;s^{-1}$
g	Reciprocal of the average fast inhibitory time constant	$500\;s^{-1}$
$C_{1}$,$C_{2}$	Average number of synaptic contacts in the excitatory feedback loop	$C_{1}=135$, $C_{2}=108$
$C_{3}$,$C_{4}$	Average number of synaptic contacts in the slow feedback inhibitory loop	$C_{3}=33.75$, $C_{4}=33.75$
$C_{5}$,$C_{6}$,$C_{7}$	Average number of synaptic contacts in the fast feedback inhibitory loop	$C_{5}=40.5$, $C_{6}=13.5$, $C_{7}=108$
$v_{0}$,$e_{0}$,r	Parameters of the nonlinear sigmoid function S	$v_{0}=6\;\mathrm{mV}$, $e_{0}=2.5\;s^{-1}$, $r=0.56\;{\mathrm{mV}}^{-1}$
μ,$\sigma^{2}$	The mean and variance of the external input	μ=90, $\sigma^{2}=30$

## Data Availability

Data are available upon request to the corresponding author. The data are not publicly available due to them containing information that could compromise the privacy of research participants.

## References

[1] Cakan C., Jajcay N., Obermayer K. (2023). Neurolib: A Simulation Framework for Whole-Brain Neural Mass Modeling. Cogn. Comput..

[2] Papadopoulos L., Lynn C.W., Battaglia D., Bassett D.S. (2020). Relations between Large-Scale Brain Connectivity and Effects of Regional Stimulation Depend on Collective Dynamical State. PLoS Comput. Biol..

[3] Buzsáki G. (2006). Rhythms of the Brain.

[4] Vecchio F., Miraglia F., Alú F., Orticoni A., Judica E., Cotelli M., Rossini P.M. (2021). Contribution of Graph Theory Applied to EEG Data Analysis for Alzheimer’s Disease Versus Vascular Dementia Diagnosis. J. Alzheimer’s Dis..

[5] Mammone N., De Salvo S., Bonanno L., Ieracitano C., Marino S., Marra A., Bramanti A., Morabito F.C. (2019). Brain Network Analysis of Compressive Sensed High-Density EEG Signals in AD and MCI Subjects. IEEE Trans. Ind. Inform..

[6] Núñez P., Poza J., Gómez C., Barroso-García V., Maturana-Candelas A., Tola-Arribas M.A., Cano M., Hornero R. (2020). Characterization of the Dynamic Behavior of Neural Activity in Alzheimer’s Disease: Exploring the Non-Stationarity and Recurrence Structure of EEG Resting-State Activity. J. Neural Eng..

[7] Köksal Ersöz E., Wendling F. (2021). Canard Solutions in Neural Mass Models: Consequences on Critical Regimes. J. Math. Neurosci..

[8] Karoly P.J., Kuhlmann L., Soudry D., Grayden D.B., Cook M.J., Freestone D.R. (2018). Seizure Pathways: A Model-Based Investigation. PLoS Comput. Biol..

[9] Kuhlmann L., Freestone D.R., Manton J.H., Heyse B., Vereecke H.E.M., Lipping T., Struys M.M.R.F., Liley D.T.J. (2016). Neural Mass Model-Based Tracking of Anesthetic Brain States. Neuroimage.

[10] Liang Z., Wang D., Jin X., Fan L., Wen X., Wei C., Li X. (2023). Tracking the Effects of Propofol, Sevoflurane and (S)-Ketamine Anesthesia Using an Unscented Kalman Filter-Based Neural Mass Model. J. Neural Eng..

[11] Lopez A., Castillo B., Medina L., Ventura C. (2014). State and Parameter Estimation of a Neural Mass Model from Electrophysiological Signals during Induced Status Epilepticus. NeuroImage.

[12] Cui D., Li H., Liu P., Gu G., Li X., Wang L., Yin S. (2022). Analysis of the Neural Mechanism of Spectra Decrease in MCI by a Thalamo-Cortical Coupled Neural Mass Model. J. Neural Eng..

[13] Freeman W.J. (1972). Linear Analysis of the Dynamics of Neural Masses. Annu. Rev. Biophys. Bioeng..

[14] Wilson H.R., Cowan J.D. (1972). Excitatory and Inhibitory Interactions in Localized Populations of Model Neurons. Biophys. J..

[15] Lopes da Silva F.H., Hoeks A., Smits H., Zetterberg L.H. (1974). Model of Brain Rhythmic Activity: The Alpha-Rhythm of the Thalamus. Kybernetik.

[16] Moustafa A.A., McMullan R.D., Rostron B., Hewedi D.H., Haladjian H.H. (2017). The Thalamus as a Relay Station and Gatekeeper: Relevance to Brain Disorders. Rev. Neurosci..

[17] Jansen B.H., Rit V.G. (1995). Electroencephalogram and Visual Evoked Potential Generation in a Mathematical Model of Coupled Cortical Columns. Rev. Neurosci..

[18] Wendling F., Bartolomei F., Bellanger J.J., Chauvel P. (2002). Epileptic Fast Activity Can Be Explained by a Model of Impaired GABAergic Dendritic Inhibition: Epileptic Activity Explained by Dendritic Dis-Inhibition. Eur. J. Neurosci..

[19] Fan X., Gaspard N., Legros B., Lucchetti F., Ercek R., Nonclercq A. (2018). Dynamics Underlying Interictal to Ictal Transition in Temporal Lobe Epilepsy: Insights from a Neural Mass Model. Eur. J. Neurosci..

[20] Arrais M., Wendling F., Modolo J. Identification of Effective Stimulation Parameters to Abort Epileptic Seizures in a Neural Mass Model. Proceedings of the 2019 41st Annual International Conference of the IEEE Engineering in Medicine and Biology Society (EMBC).

[21] Köksal Ersöz E., Modolo J., Bartolomei F., Wendling F. (2020). Neural Mass Modeling of Slow-Fast Dynamics of Seizure Initiation and Abortion. PLoS Comput. Biol..

[22] Ursino M., Cona F., Zavaglia M. (2010). The Generation of Rhythms within a Cortical Region: Analysis of a Neural Mass Model. Neuroimage.

[23] Leon P., Knock S., Woodman M., Domide L., Mersmann J., McIntosh A., Jirsa V. (2013). The Virtual Brain: A Simulator of Primate Brain Network Dynamics. Front. Neuroinform..

[24] Honey C.J., Sporns O., Cammoun L., Gigandet X., Thiran J.P., Meuli R., Hagmann P. (2009). Predicting Human Resting-State Functional Connectivity from Structural Connectivity. Proc. Natl. Acad. Sci. USA.

[25] Schirner M., McIntosh A.R., Jirsa V., Deco G., Ritter P. (2018). Inferring Multi-Scale Neural Mechanisms with Brain Network Modelling. eLife.

[26] Giannakakis E., Han C.E., Weber B., Hutchings F., Kaiser M. (2020). Towards Simulations of Long-Term Behavior of Neural Networks: Modeling Synaptic Plasticity of Connections within and between Human Brain Regions. Neurocomputing.

[27] Endo H., Hiroe N., Yamashita O. (2020). Evaluation of Resting Spatio-Temporal Dynamics of a Neural Mass Model Using Resting fMRI Connectivity and EEG Microstates. Front. Comput. Neurosci..

[28] De Haan W., Van Straaten E.C.W., Gouw A.A., Stam C.J. (2017). Altering Neuronal Excitability to Preserve Network Connectivity in a Computational Model of Alzheimer’s Disease. PLoS Comput. Biol..

[29] Taylor P.N., Kaiser M., Dauwels J. (2014). Structural Connectivity Based Whole Brain Modelling in Epilepsy. J. Neurosci. Methods.

[30] Hutchings F., Han C.E., Keller S.S., Weber B., Taylor P.N., Kaiser M. (2015). Predicting Surgery Targets in Temporal Lobe Epilepsy through Structural Connectome Based Simulations. PLoS Comput. Biol..

[31] Kunze T., Hunold A., Haueisen J., Jirsa V., Spiegler A. (2016). Transcranial Direct Current Stimulation Changes Resting State Functional Connectivity: A Large-Scale Brain Network Modeling Study. Neuroimage.

[32] Schirner M., Rothmeier S., Jirsa V.K., McIntosh A.R., Ritter P. (2015). An Automated Pipeline for Constructing Personalized Virtual Brains from Multimodal Neuroimaging Data. Neuroimage.

[33] Desikan R.S., Ségonne F., Fischl B., Quinn B.T., Dickerson B.C., Blacker D., Buckner R.L., Dale A.M., Maguire R.P., Hyman B.T. (2006). An Automated Labeling System for Subdividing the Human Cerebral Cortex on MRI Scans into Gyral Based Regions of Interest. Neuroimage.

[34] Fischl B. (2012). FreeSurfer. Neuroimage.

[35] Xia M., Wang J., He Y. (2013). BrainNet Viewer: A Network Visualization Tool for Human Brain Connectomics. PLoS ONE.

[36] Watts D.J., Strogatz S.H. (1998). Collective Dynamics of ‘Small-World’ Networks. Nature.

[37] Liao X., Vasilakos A.V., He Y. (2017). Small-World Human Brain Networks: Perspectives and Challenges. Neurosci. Biobehav. Rev..

[38] Carvalho V.R., Moraes M.F.D., Cash S.S., Mendes E.M.A.M. (2021). Active Probing to Highlight Approaching Transitions to Ictal States in Coupled Neural Mass Models. PLoS Comput. Biol..

[39] Dallmer-Zerbe I., Jajcay N., Chvojka J., Janca R., Jezdik P., Krsek P., Marusic P., Jiruska P., Hlinka J. (2023). Computational Modeling Allows Unsupervised Classification of Epileptic Brain States across Species. Sci. Rep..

[40] Prathaban B.P., Rajendran S., Ganeshkumar N., Gayatri M., Chembian W.T. (2023). Interpretation of Seizure Dynamics Using Fuzzy-Based Neural Computational Modelling. Soft Comput..

[41] Hebbink J., van Gils S.A., Meijer H.G.E. (2020). On Analysis of Inputs Triggering Large Nonlinear Neural Responses Slow-Fast Dynamics in the Wendling Neural Mass Model. Commun. Nonlinear Sci. Numer. Simul..

